# Statistical approaches to temporal and spatial autocorrelation in resting-state functional connectivity in mice measured with optical intrinsic signal imaging

**DOI:** 10.1117/1.NPh.9.4.041405

**Published:** 2022-03-14

**Authors:** Brian R. White, Claudia Chan, Simon Vandekar, Russell T. Shinohara

**Affiliations:** a University of Pennsylvania, Children’s Hospital of Philadelphia, Perelman School of Medicine, Division of Cardiology, Department of Pediatrics, Philadelphia, Pennsylvania, United States; b Vanderbilt University, Department of Biostatistics, Nashville, Tennessee, United States; c University of Pennsylvania, Perelman School of Medicine, Department of Biostatistics, Epidemiology, and Informatics, Philadelphia, Pennsylvania, United States; d University of Pennsylvania, Center for Biomedical Image Computing and Analysis, Department of Radiology, Philadelphia, Pennsylvania, United States; e University of Pennsylvania, Penn Statistics in Imaging and Visualization Endeavor, Department of Biostatistics, Epidemiology, and Informatics, Philadelphia, Pennsylvania, United States

**Keywords:** functional neuroimaging, optical intrinsic signal, statistics, resting-state, functional connectivity, autocorrelation

## Abstract

**Significance:**

Resting-state functional connectivity imaging in mice with optical intrinsic signal (OIS) imaging could provide a powerful translational tool for developing imaging biomarkers in preclinical disease models. However, statistical interpretation of correlation coefficients is hampered by autocorrelations in the data.

**Aim:**

We sought to better understand temporal and spatial autocorrelations in optical resting-state data. We then adapted statistical methods from functional magnetic resonance imaging to improve statistical inference.

**Approach:**

Resting-state data were obtained from mice using a custom-built OSI system. The autocorrelation time was calculated at each pixel, and z scores for correlation coefficients were calculated using Fisher transforms and variance derived from either Bartlett’s method or xDF. The significance of each correlation coefficient was determined through control of the false discovery rate (FDR).

**Results:**

Autocorrelation was generally even across the cortex and parcellation reduced variance. Correcting variance with Bartlett’s method resulted in a uniform reduction in z scores, with xDF preserving high z scores for highly correlated data. Control of the FDR resulted in reasonable thresholding of the correlation coefficient matrices. The use of Bartlett’s method compared with xDF results in more conservative thresholding and fewer false positives under null hypothesis conditions.

**Conclusions:**

We developed streamlined methods for control of autocorrelation in OIS functional connectivity data in mice, and Bartlett’s method is a reasonable compromise and simplification that allows for accurate autocorrelation correction. These results improve the rigor and reproducibility of functional neuroimaging in mice.

## 1 Introduction

All functional neuroimaging systems (whether assessing responses to external stimuli or the resting state) face the challenge of separating signal from noise. This determination of statistical significance is complicated by autocorrelation in the signals. Autocorrelation takes two forms. First, the time series of neuronal or hemodynamic activity are often temporally autocorrelated. Second, nearby pixels or voxels are spatially autocorrelated. In both cases, autocorrelation arises from underlying physiology, the mechanics of the neuroimaging system, and signal processing choices (e.g., temporal filtering or spatial smoothing). Functional information is often extracted via a correlation coefficient (either against a task-based regressor or against a “seed” region in resting-state functional connectivity). The raw values from this statistical test need to be adjusted to determine a more appropriate threshold. Many statistical approaches for alleviating these issues have been developed for functional magnetic resonance imaging (fMRI). However, few of these methods have been adapted for analysis of optical neuroimaging data in mice. Our goal in this manuscript is to adapt methods from fMRI to the particular statistical needs of resting-state functional connectivity data obtained in mice using optical intrinsic signal (OIS) imaging.

OIS [also referred to as intrinsic optical signal (IOS) and widefield optical imaging (WOI)] uses changes in the absorption of visible light to measure changes in the concentrations of oxy- and deoxyhemoglobin.^1^^–^^3^ Functional neuroimaging with OIS through neurovascular coupling accesses neuronal activity in a manner akin to the blood oxygen level-dependent fMRI contrast.^4^^,^^5^ With intact-skull cranial windows,^6^ it is possible to image the dorsal convexity of the mouse brain and visualize multiple canonical resting-state networks.^7^^,^^8^ Such methods provide a link between advanced neuroimaging diagnostic techniques developed in humans and mouse models of disease.^9^^–^^11^ However, the development of statistical techniques for OIS data has been slower than that for fMRI or human optical neuroimaging methods.

Thus, in this paper, we adapted fMRI techniques for the statistical analysis of resting-state functional connectivity OIS data to account for temporal and spatial autocorrelation. Our focus is the determination of which correlations within a given mouse are significant rather than searching for differences between two experimental populations. To address temporal autocorrelation, we generally follow the approach of Afyouni et al.^12^ and compare three methods: (1) naïve (i.e., no correlation), (2) “Bartlett’s method,” and (3) xDF (a new method proposed by Afyouni et al. as a “variance estimator for Pearson’s correlation that imposes no assumptions aside from stationarity”). Accounting for spatial autocorrelation is a more contentious subject in fMRI, with many authors attempting to correct the familywise error rate (FWE).^13^ A common method to account for spatial dependence in neuroimaging data is to use Gaussian random field theory (RFT).^14^^,^^15^ However, we believe that the random field approach suffers from theoretical drawbacks because it relies on assumptions that may not be satisfied for OIS data. These concerns will be discussed further in Sec. 4. Additionally, to avoid the well-documented conservatism of classical FWE correction methods, we propose controlling the false discovery rate (FDR).^16^^–^^18^ The success of these methods is demonstrated using functional connectivity data obtained in healthy mice.

## 2 Methods

### 2.1 Imaging system, Data Acquisition, and Preprocessing

**Fig. 1:**
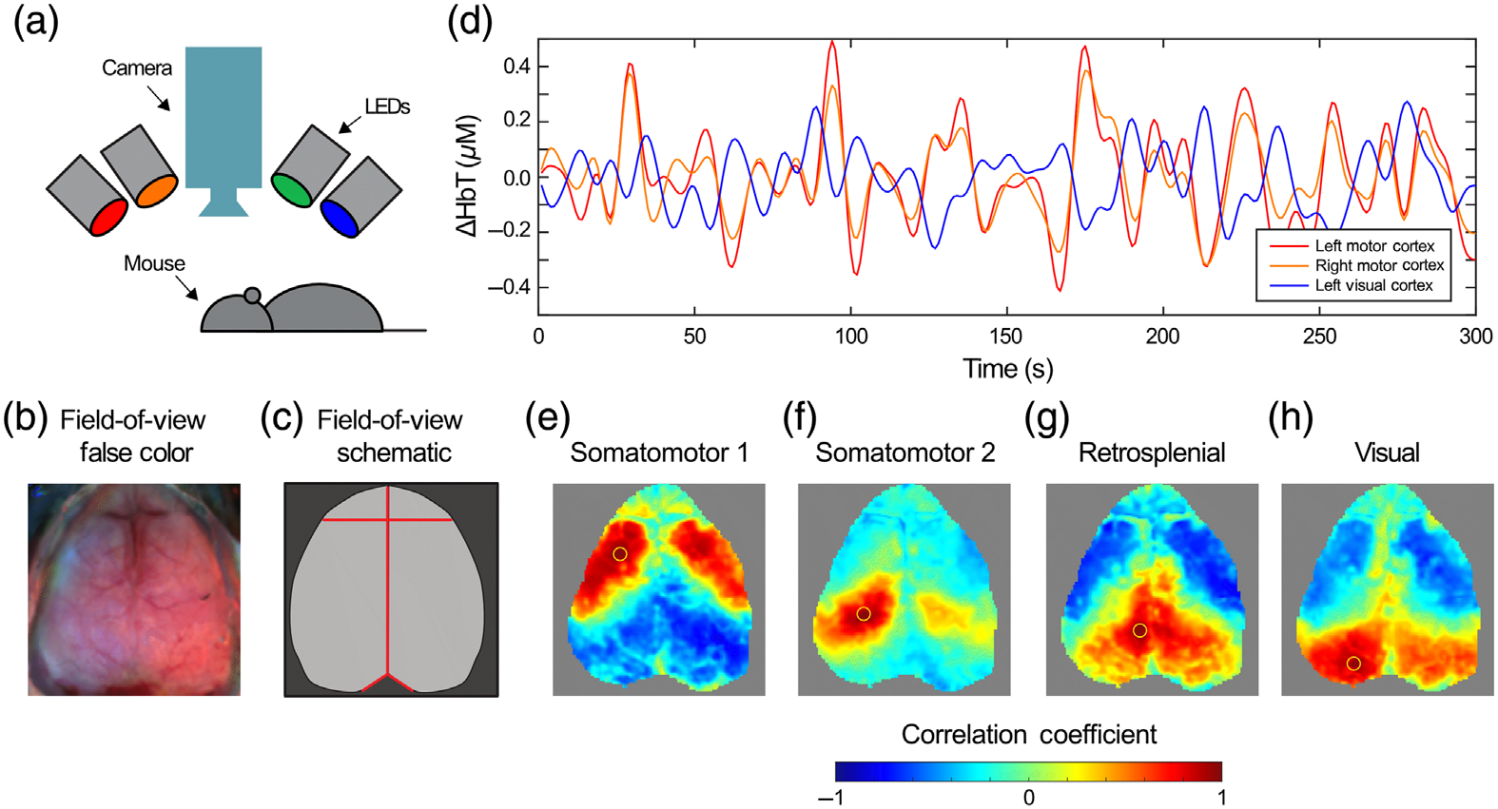
System field-of-view and canonical resting-state functional connectivity networks. (a) System diagram showing the LEDs and camera over the dorsal surface of the mouse brain. (b) False color image and schematic (c) of the mouse brain from above as seen by the system. Around the perimeter is scalp and skull (dark gray in the schematic). The segmented brain is central (light gray in the schematic). Landmarks for atlas registration are shown in red. (d) Example hemodynamic time series of changes in total hemoglobin concentration in the mouse brain. Note that the right and left motor cortices are highly correlated (r=0.93) and the left motor and visual cortices are uncorrelated (r=−0.46). (e)–(h) Exemplary seed-based resting-state functional connectivity maps (seeds highlighted in yellow) showing canonical networks.

All mouse procedures were approved by the Institutional Animal Care and Use Committee of the Children’s Hospital of Philadelphia. Data were obtained from 13 mice with a median of five 5-min runs per mouse (range 2 to 25). The imaging system and procedures have been described previously.^7^^,^^19^ Briefly, mice were anesthetized with a mixture of ketamine (100 mg/kg) and xylazine (10 mg/kg) before being imaged with the OIS system. The OIS system [Fig. 1(a)] consisted of four, temporally multiplexed light emitting diodes (LEDs, M470L3, M530L3, M590L3, and M625L3, Thorlabs) and a camera (iXon 887 or Zyla 4.2, Andor Technologies), which acquired sequential images (128×128  pixels) for an overall frame rate of 30 Hz (note that, of the 112 total scans, 25 were performed using the newer Zyla imaging system). The nominal central wavelengths for the LEDs were 470, 530, 590, and 625 nm, respectively; however, individualized spectra for each LED were measured as previously described.^19^ The field-of-view is the dorsal surface of the mouse brain [Figs. 1(b) and 1(c)].

**Fig. 2:**
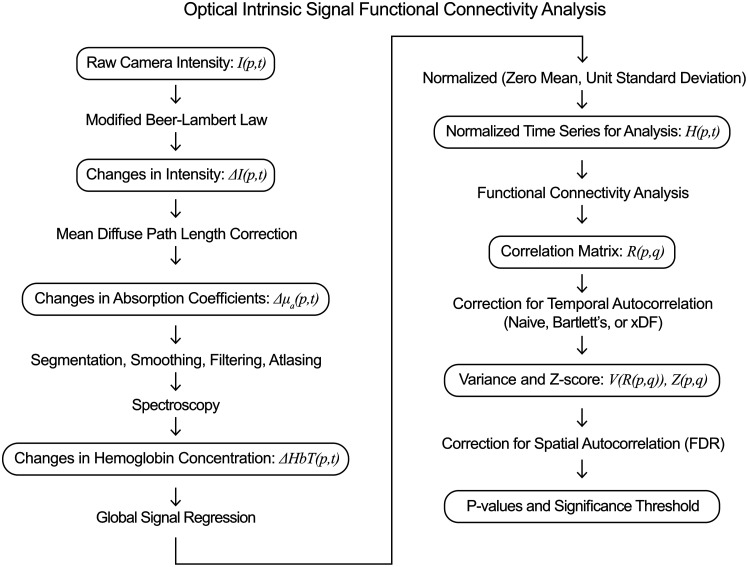
Flowchart of data analysis, including definitions of the variables used in this manuscript. Following prior methods, raw camera data are converted to a normalized time series of changes in total hemoglobin at each pixel p at time t: H(p,t). Functional connectivity correlation analysis generates a correlation matrix of correlations between each pair of pixels p and q: R(p,q). The new analysis methods of this manuscript involve (1) analysis of temporal autocorrelation to calculate an appropriate variance of each correlation coefficient V(p,q) and resulting z score Z(p,q) and (2) the correction of spatial autocorrelation using control of the FDR to find an appropriate p-value threshold for significance.

The initial analysis follows previously published methods (Fig. 2).^7^^,^^19^^–^^21^ Changes in intensity at each pixel in each color illumination were converted to changes in absorption coefficients using a modified Beer–Lambert lab approach and mean diffuse pathlengths calculated using the analytical solution to the diffusion approximation of the radiative transfer equation in a semi-inﬁnite geometry.^19^ The brain was segmented using a combination of automatic and manual methods.^20^ Images were smoothed with a 5×5  pixel Gaussian filter (standard deviation: 1 pixel), and data were aligned to the Paxinos atlas by aligning landmarks in the images to standard coordinates using an affine transform.^21^ These absorption changes were filtered to 0.01 to 0.1 Hz before spectroscopy was performed using wavelength-censored methods;^19^ all data shown use changes in the total hemoglobin contrast (ΔHbT), as this has been shown to have the highest signal-to-noise.^8^^,^^19^ Data were downsampled to 1 Hz.

After preprocessing, the data for each scan consist of time series of changes in total hemoglobin for each pixel Δ[HbT](p,t), where p is an index of all pixels in the field of view (1,…,P) and t is an index of time (1,…,T). Analysis was performed only on those pixels within the brain segmentation that passed the quality control thresholds. These pixels are defined by a brain mask B(p) consisting of ones (pixels that are analyzed) and zeroes (excluded pixels).

### 2.2 Functional Connectivity Analysis

For each run, the global signal was calculated by averaging over all pixels within the brain mask, and this was regressed from all pixels. The time series were then normalized to zero mean and unit standard deviation. Let H(p,t) be the normalized change in total hemoglobin at pixel p and time t. Let R(p,q)=ρ(H(p,t),H(q,t)) be the Pearson’s correlation coefficient between the time courses and pixels p and q [Fig. 1(d)]. Using these methods, canonical resting-state functional connectivity networks in the mouse are visualized [Figs. 1(e)–1(h)].

As Pearson’s correlation coefficients are not normally distributed, prior to statistical analysis, they are commonly converted to *z* scores via the Fisher transformation,^12^^,^^22^ F(p,q)=arctanh(R(p,q)), and

$$Z(p,q)=\frac{F(p,q)}{\sqrt{V(F(p,q))}}=\frac{F(p,q)}{\sqrt{V(R(p,q))(1-R(p,q{)}^{2}{)}^{-2}}},$$

where Z(p,q) is the z score of the Pearson’s correlation coefficient and V is the variance. The resulting p-value is then obtained using the two-tailed normal cumulative distribution function.

In the absence of autocorrelation, $V(R(p,q))\approx (1-R(p,q{)}^{2}{)}^{2}/(T-3)$. Thus in this situation, Z(p,q) reduces to

$$Z(p,q)=\sqrt{T-3}\;F(p,q).$$

### 2.3 Temporal Autocorrelation

OIS data, like that from fMRI, are temporally autocorrelated, and each point is not an independent observation. This autocorrelation results in an increase in the variance of the Pearson’s correlation coefficient. The resulting uncorrected (naïve) z score is then spuriously elevated, and the distribution of z scores is no longer normal, violating the assumptions that allow the z score to be converted to a p-value.

In resting-state functional connectivity analysis, the most common method to account for temporal autocorrelation is often referred to as “Bartlett’s method.”^23^ Here the variance is adjusted using “effective degrees of freedom,” T^ such that

V(R(p,q))=(1−R(p,q)2)2T^.

T^ is defined using the global average of the autocorrelation time:

T^=T⟨τ(p)⟩.

The autocorrelation time is calculated for each pixel in each 5-min run as^12^

$$\tau (p)=\sum_{i=0}^{T-1}\rho (H(p,t),H(p,t+i){)}^{2}.$$

In practice, this sum is truncated or tapered to avoid spurious correlations at long delay times. We use Tukey tapering with a maximum lag of $2\sqrt{T}.$ However, the Bartlett approximation for the variance under autocorrelation is only accurate under the null hypothesis (R(p,q)=0) and when there is little spatial variation in τ.

Due to these limitations, Afyouni et al.^12^ developed a more general formulation for the variance of Pearson’s correlation coefficient, which they termed xDF. Assuming stationary covariance (i.e., that the autocorrelation does not change with time), then the cross correlation is defined as

$$R_{c}(p,q)=\rho (H(p,t),H(q,t+c)),$$

such that $R_{0}(p,q)$ is simply the correlation coefficient. Then xDF is given by

V(R(p,q))=1T2[(T−2)(1−R0(p,q)2)2+R0(p,q)2∑k=1T(T−2−k)(Rk(p,p)2+Rk(q,q)2+Rk(p,q)2R−k(p,q)2)−2R0(p,q)∑k=1T(T−2−k)(Rk(p,p)+Rk(q,q))(Rk(p,q)+R−k(p,q))+2∑k=1T(T−2−k)(Rk(p,p)Rk(q,q)+Rk(p,q)R−k(p,q))],

which is Eq. (2) in Ref. 12. (Again, in practice, the sums run to a maximum of $2\sqrt{T}$ to avoid spurious effects at long times.) Note that we put a floor on the xDF variance, such that V(R(p,q)) is only allowed to be as low as the naïve variance $V(R(p,q))=(1-R(p,q{)}^{2}{)}^{2}/(T-3)$.

### 2.4 Assessing the Effect of Temporal Autocorrelation

First, we assessed how temporal autocorrelation varied across the imaged portion of the mouse brain. We calculated τ at each pixel in each run to visualize its spatial variance. We then calculated the standard deviation in τ across all of the mice in the study. We then averaged the OIS hemodynamic signals within regions of interest as defined by the Paxinos histological atlas of the mouse brain.^24^ We then repeated the above analyses to determine the spatial and interscan variance in τ for these averaged time series. The autocorrelation time was compared with the characteristic length (square root of the area) of each parcel of the Paxinos atlas via linear regression.

We then converted correlation coefficients to z scores using each of the above three variance formulas (naïve, Bartlett’s method, and xDF). First, null hypothesis data (all correlation coefficients expected to be 0) were generated by correlating every pixel from one mouse against data obtained from a second mouse. Data with true correlations (alternative hypothesis) were simply obtained from the usual procedure of correlating time series obtained within the same run.

We compared the resulting z scores by plotting the z scores from Bartlett’s method and xDF against the original naïve *z* scores. Normality of the z score distributions (for the null hypothesis data) was assessed using the Kolmogorov–Smirnov statistic. Afyouni et al.^12^ predicted that the length of the time series affects how each variance estimate results in false positives; so we performed this analysis both with single runs (of 5 min) and after concatenating 5 runs in the same mice (for 25 min of total data).

### 2.5 Computation Time

All computations were performed using MATLAB 2020a on a server with 20 CPUs and 160 GB of RAM. To assess computational efficiency, during the above experiments, the time it took to calculate V(R(p,q)) by both Bartlett’s method and xDF was measured using the tic and toc functions in MATLAB. Although open-source code to calculate xDF was made available by Afyouni et al., that code could not be used without modification, as the number of pixels in the OIS experiment is high enough that attempting to calculate the full cross correlation matrix using a single-matrix operation exceeds the allowable maximum variable size in MATLAB. So the use of multiple for loops is necessary, which lengthens the processing time. No parallel processing was used due to complexities of the cross correlation matrices. We thus recognize that the calculated times are a worst-case scenario for xDF.

### 2.6 Spatial Autocorrelation and the False Discovery Rate

A common way to determine statistical significance is through control of the type I error, with a threshold of p<0.05 being common. However, a typical resting-state functional connectivity study may involve thousands of comparisons between different brain regions. As a typical mouse OIS brain segmentation includes 10,000 pixels, even if the null hypothesis were always true, then 500 pixels would be judged significant by chance alone. Corrections to the p-value threshold that control the FWE, such as the Bonferroni correction, are overly conservative in the face of spatial autocorrelation. Commonly, functional neuroimaging studies attempt to adjust for the type I error using RFT.^13^ However, these methods rely on assumptions that may not be met in resting-state functional connectivity data, especially with optical methods; we explore these problems in Sec. 4.

We chose to control the FDR because it results in less conservative inference. Rather than controlling false positives as a percent of total pixels, FDR methods accept that false positives may occur but seek to limit the average number of false positives as a percent of all positive pixels. Let N be the total number of pixels for which the null hypothesis is rejected and f be the number of false positives; then the FDR is defined as the expected value of f/N. Using the Benjamini–Hochberg algorithm,^25^ we can guarantee that for any threshold γ, the FDR ≤γ. In particular, we use the Benjamini–Yekutieli extension of the Benjamini–Hochberg method to account for the presence of both positive and negative correlations.^17^^,^^26^

1. Rank order all p-values from the smallest to the largest, denoted as $p_{1},\ldots ,p_{N}$.
2. Let k denote the largest index that satisfies $p_{k}<\frac{k}{N}\frac{\gamma }{\Sigma_{j=1}^{N}1/j}$.
3. Then reject the null hypotheses for $p_{1},\ldots ,p_{k}$, which controls the FDR at level γ.

It is important to note that this algorithm is performed once for the entire correlation matrix and not individually on each map from a single seed (i.e., each row or column of the correlation matrix). Furthermore, note that the correlation matrix is symmetric. Thus the only p-values considered are those in the lower triangle of the correlation matrix (not including the main diagonal, which necessarily consists solely of correlation values of 1).

We demonstrate the use of this algorithm to determine the threshold for statistical significance using resting-state functional connectivity data, as previously (Sec 2.2). The p-values used were generated with both Bartlett’s method and xDF. First, we used data for which the null hypothesis is true by considering correlations between two separate runs or two different mice to calculate the percentage of false positives generated with various FDR thresholds. The allowed FDR was varied between $10^{-5}$ and $10^{-2}$, and the percentage of positive correlations was calculated for all 6216 possible combinations of runs. The median across runs for all FDRs was determined.

We then used resting-state correlations within the same run to assess how FDR control provides a threshold for seed-based functional connectivity analysis. We examined how the thresholded maps differed when using p-values from Bartlett’s method or xDF using a fixed FDR. Finally, we assessed how sensitive the maps were to the choice of FDR. The FDR was varied from $10^{-5}$ to $10^{-2}$, and we noted at which level a pixel lost statistical significance.

## 3 Results

### 3.1 Autocorrelation Time

**Fig. 3:**
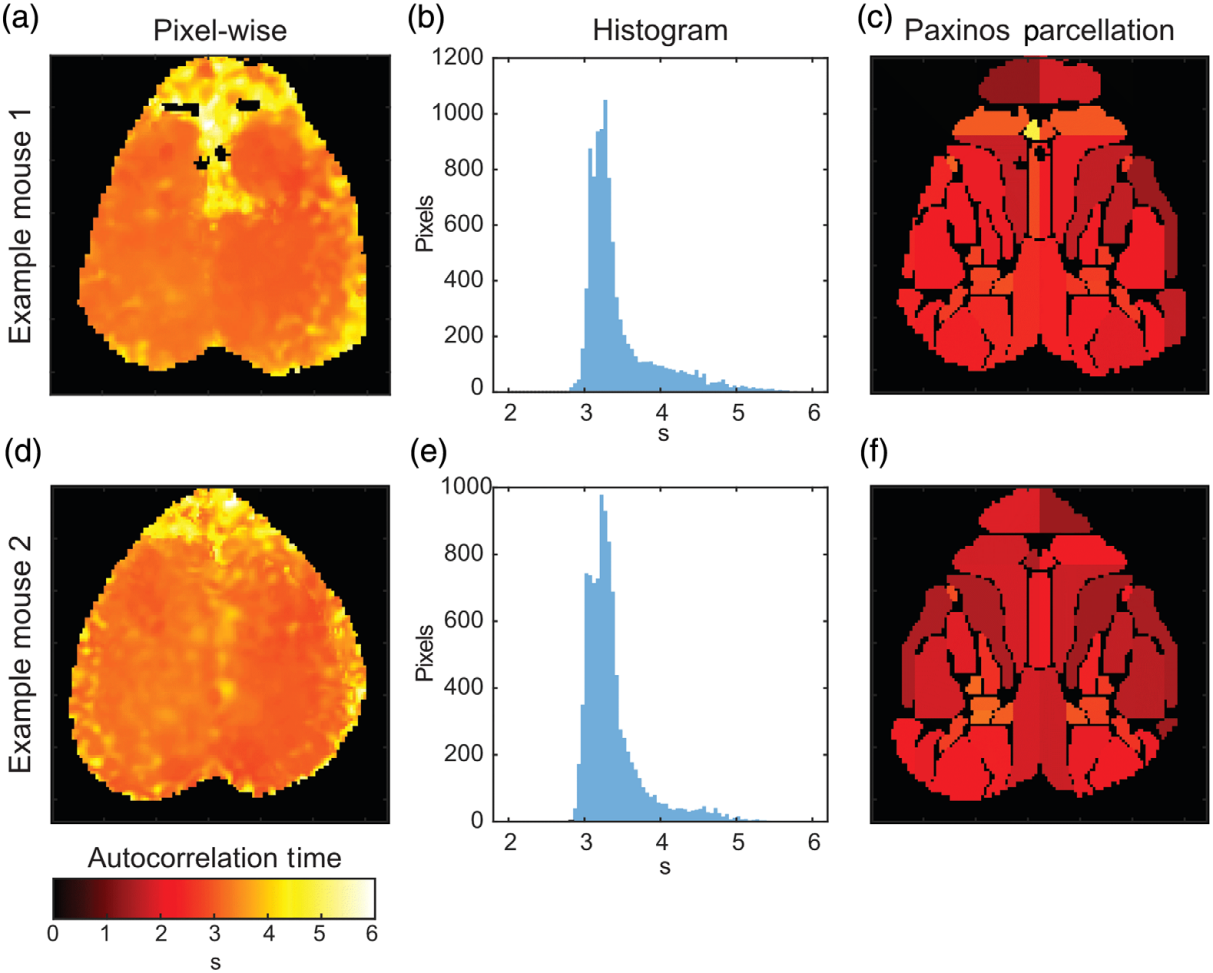
Example of autocorrelation time maps across the mouse cortex. (a) The autocorrelation in each pixel. (b) Histogram of the data in (a) demonstrating that the majority of pixels have an autocorrelation time of 3 to 3.5 s with a long tail of pixels with higher correlation times. The median autocorrelation time was 3.28 s (interquartile range: 3.16 to 3.54). (c) Autocorrelation times for hemodynamic traces for regions of interest defined by the Paxinos histologic atlas of the mouse brain. The median autocorrelation time within parcels was 2.53 s (interquartile range: 2.05 to 2.93). (d)–(f) Similar results for (a)–(c) for a second example mouse. For the unaveraged data, the median autocorrelation time was 3.27 s (interquartile range: 3.12 to 3.43). For spatially averaged data, the median autocorrelation time was 2.08 s (interquartile range: 1.78 to 2.62).

We first assessed variation in the autocorrelation time, spatially across the mouse brain and across mice. Generally, the autocorrelation time was stable across the cerebral cortex [Figs. 3(a) and 3(d)] between 3 and 3.5 s [Figs. 3(b) and 3(e)]. The autocorrelation times tended to be longer in areas of lower signal quality such as in the olfactory bulb, along the anterior midline (underneath a venous sinus), and at the periphery of the brain segmentation, with some pixels having an autocorrelation time near 6 s. Unlike the finding of Afyouni et al. in the human brain, averaging the hemodynamic time course across regions of interest defined by the Paxinos atlas reduced variation in the autocorrelation time across the cerebral cortex [Figs. 3(c) and 3(f)]. Parcellation also lowered the autocorrelation time, especially in the areas of the brain with lower signal-to-noise around the periphery.

**Fig. 4:**
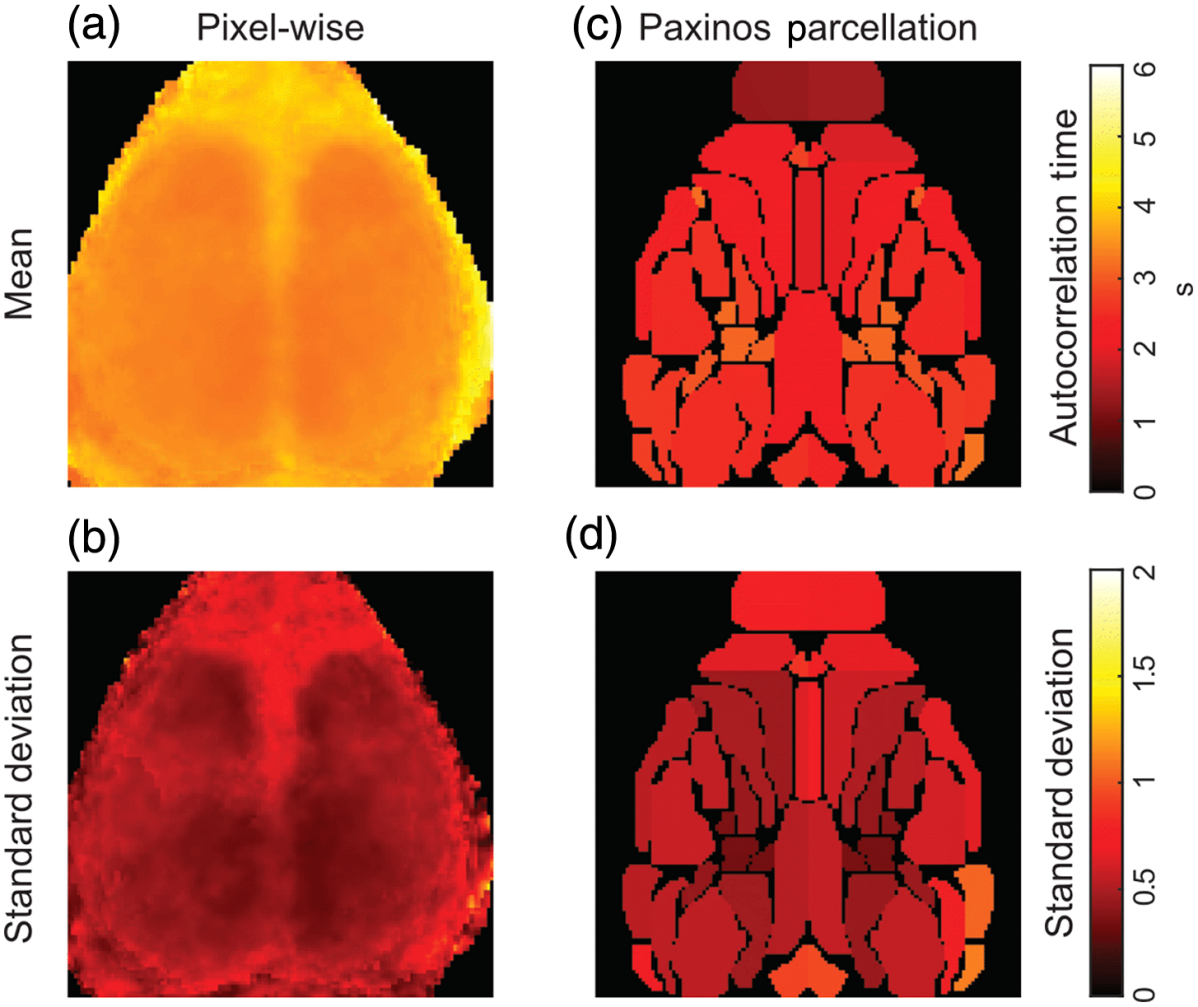
Autocorrelation maps in the mouse cortex averaged across mice. (a) Averaged autocorrelation times. (b) The standard deviation of the autocorrelation time in each pixel across mice and runs. (c), (d) The same analysis as (a) and (b) considering averaged signals in each region of interest from the Paxinos atlas.

Similar findings were seen when the maps of the autocorrelation time were averaged across mice. The autocorrelation time was even across the cortex with higher values in the olfactory bulb, midline, and periphery [Fig. 4(a)]. These areas of higher autocorrelation time were also regions of higher standard deviation across mice [Fig. 4(b)]. Again, parcellation with the Paxinos atlas resulted in lower autocorrelations times. With the pixel-wise data, the median autocorrelation time across the brain was 3.46 s (interquartile range: 3.38 to 3.68). After averaging within ROIs, the mean autocorrelation time across the brain decreased to 2.58 s (interquartile range: 2.20 to 2.97 s). The characteristic length (in pixels) of each parcel was inversely related to the autocorrelation time in the parcel (r=0.72, slope=−0.058). Although not formally statistically tested, we did not note any major differences in the autocorrelation time measured by the two systems (iXon and Zyla cameras).

### 3.2 Temporal Autocorrelation

**Fig. 5:**
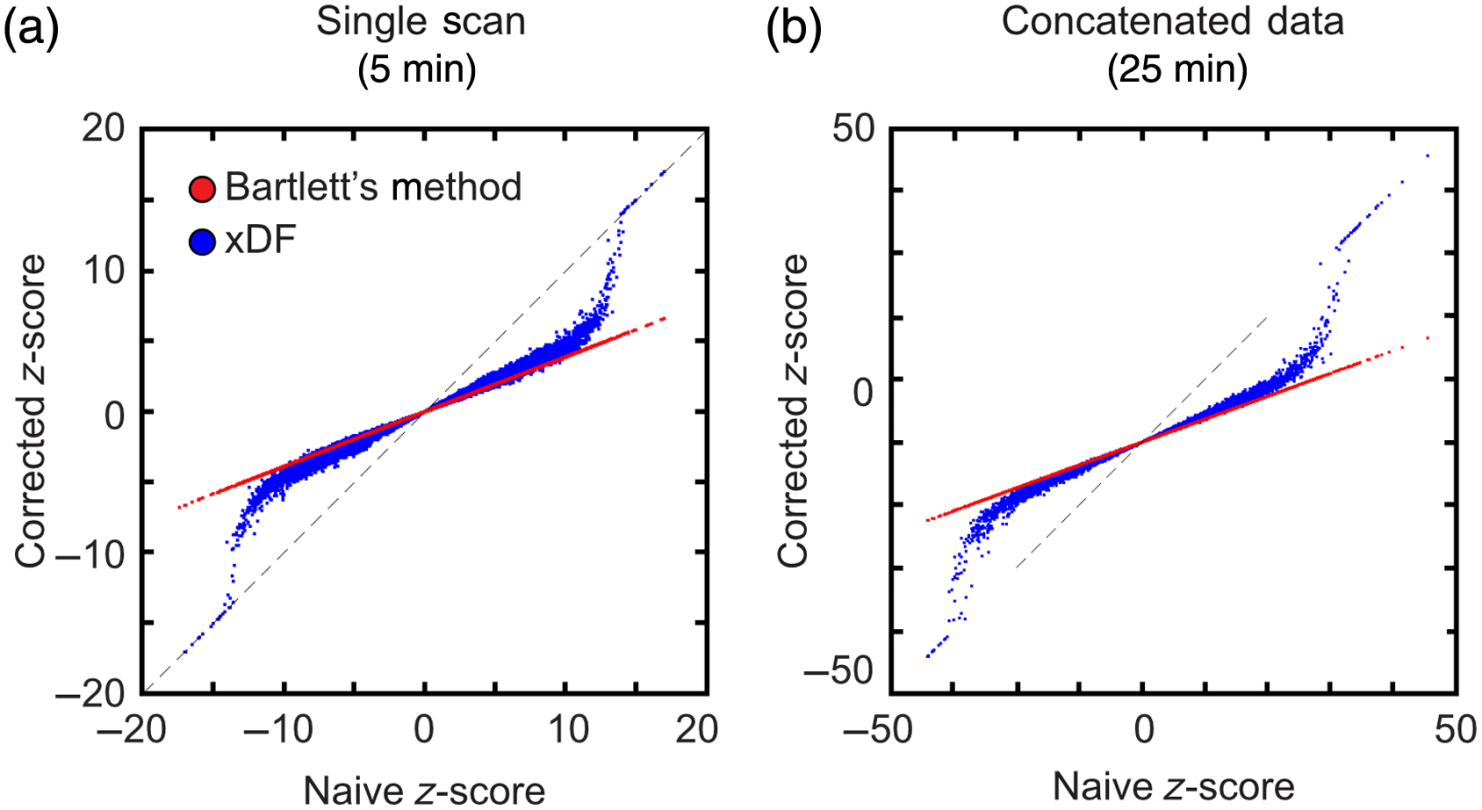
Comparison of Z scores calculated from null hypothesis data using different corrections for temporal autocorrelation. Z scores for both Bartlett’s method and xDF are compared with naïve z scores (the x axis). Bartlett’s method results in a uniform reduction in Z score, whereas xDF preserves high z scores for highly correlated data. (a) Data from two 5-min runs and (b) data from correlating 25 min of concatenated data.

Next, we compared the effect of different compensations for temporal autocorrelation on the z scores for resting-state functional connectivity matrices. To generate data under the null hypothesis, we correlated data from one mouse to data from another mouse; both scans were 300 s long sampled at 1 Hz. Z scores were calculated using naïve, Bartlett’s method, and xDF [Fig. 5(a)]. In this mouse, Bartlett’s method resulted in an effective degrees of freedom of 45.1. We see that, as expected, Bartlett’s method reduces z scores by this fixed ratio. xDF is similar to Bartlett’s method at low correlation values, with the assumptions in Bartlett’s method being most accurate, whereas at higher correlation values, xDF results in higher z scores. As in Afyouni et al., Bartlett’s method results in the lowest Kolmogorov–Smirnov statistic of 0.0326 compared with 0.238 for naïve and 0.0720 for xDF, indicating that the Bartlett’s method data most closely followed a normal distribution.

As Afyouni et al. found that the performance advantage of xDF over Bartlett’s method improved with the increasing length of the time series involved, the above analysis was repeated using null data generated by correlating 25 min (5 concatenated 5-min runs) from the same two mice. The results are similar [Fig. 5(b)]. Bartlett’s method had the lowest (most normal) Kolmogorov–Smirnov statistic of 0.060 compared with 0.091 from xDF and 0.266 from naïve. When analyzing high-resolution data, the calculation of variance with Bartlett’s method took a mean of 0.80 s (range 0.73 to 0. 84 s) versus a mean of 17,401 s (about 4.8 h, range 14,541 to 20,037 s) for xDF.

### 3.3 Spatial Autocorrelation

**Fig. 6:**
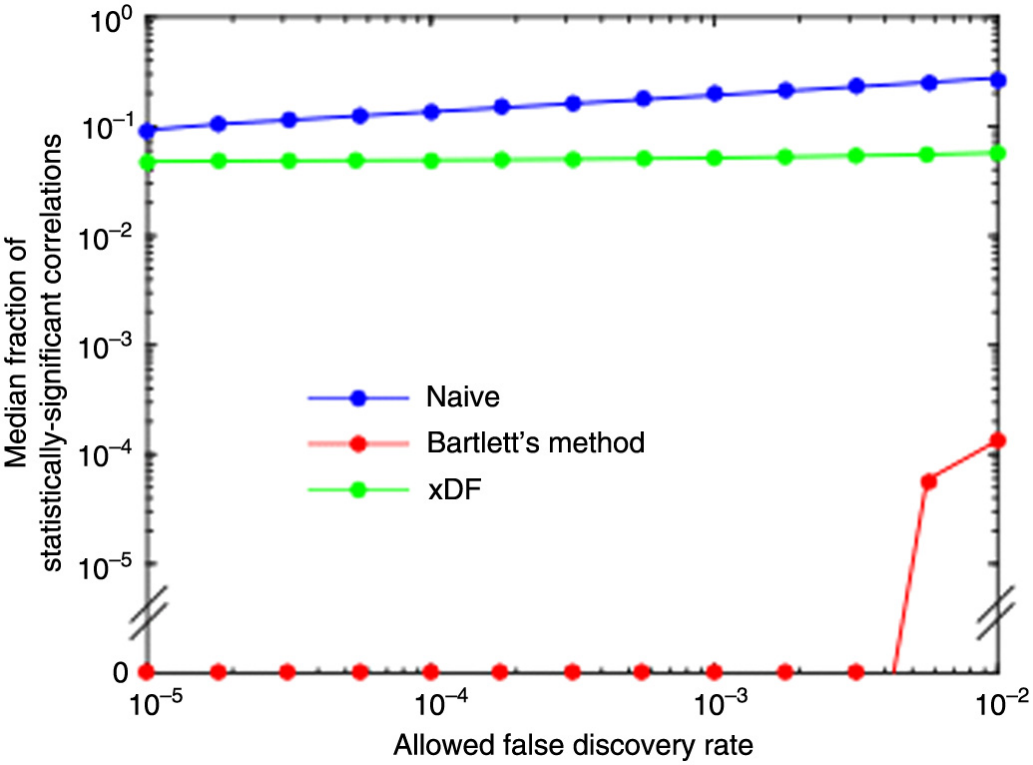
The experimental false positive rate of OIS seed-based functional connectivity data under null hypothesis conditions using different variance corrections for temporal autocorrelation and varying the FDR threshold in the Benjamini–Yekutieli procedure.

First, we again examined the null hypothesis data generated by correlated data from different runs against each other. We compared all 6216 possible combinations of two runs. The number of significant correlations was calculated for each variance correction and for a range of FDRs; the percent of significant correlations was averaged across all comparisons. In this analysis, all of these significant correlations are false positives. First, note that naïve control of the family-wise error rate at 0.05 (|z|>2) results in a substantial number of false positives. With naïve correlation statistics, on average, 67.5% of the correlations from null hypothesis data are significant, compared with 28.0% with Barlett’s method and 38.3% with xDF. Using FDR control resulted in a substantially smaller fraction of correlations being judged significant (Fig. 6). Varying the allowed FDR (from $10^{-5}$ to $10^{-2}$), we found that, with naïve variance correction, even with an FDR of 10^−5^, a median of 9.2% of correlations were still considered statistically significant. Similarly, with this FDR and xDF variance, a median of 4.5% of all correlations were still considered significant. Conversely, using Bartlett’s method, using FDRs below $3.2\times 10^{-3}$, resulted in a median of zero correlation being significant across null hypothesis pairs of studies. At all FDR thresholds, Bartlett’s method was much more conservative than xDF in null hypothesis conditions (Fig. 6).

**Fig. 7:**
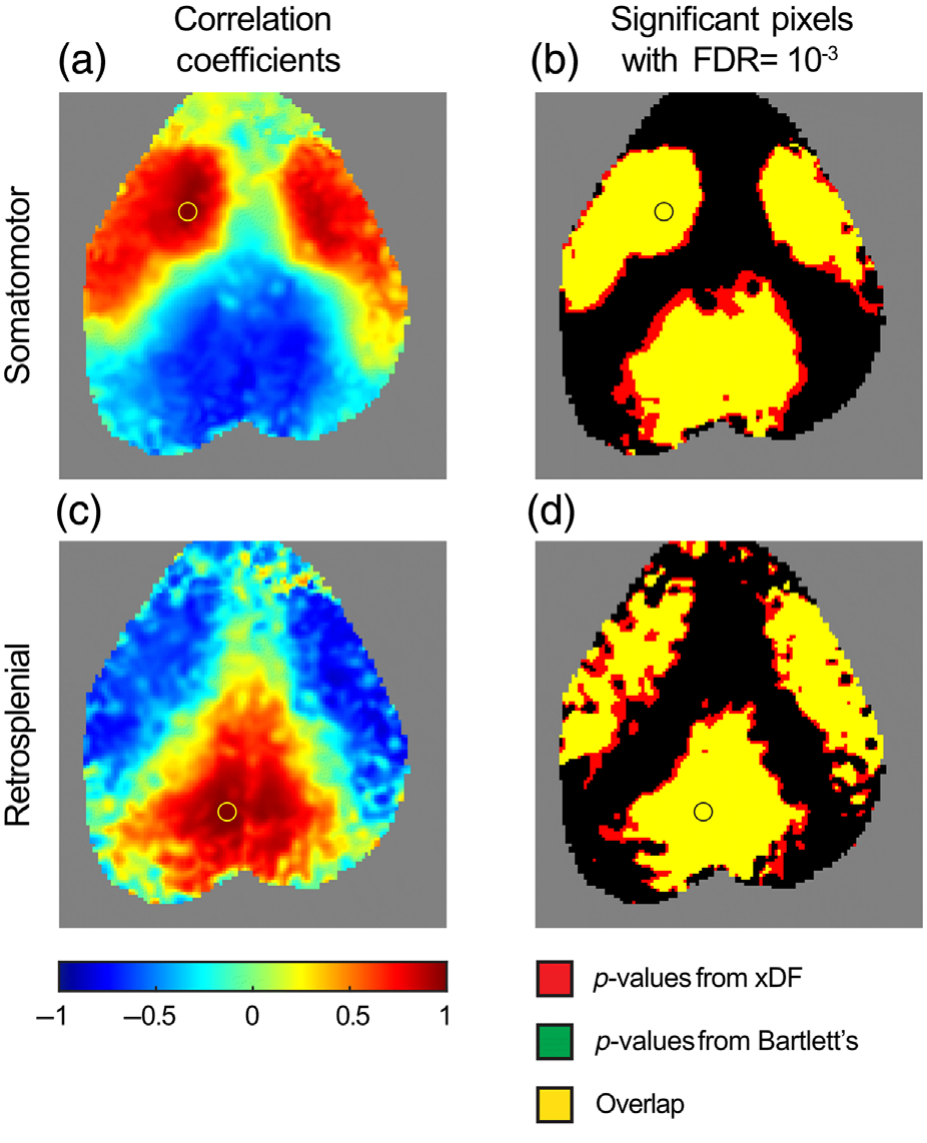
Thresholding correlation matrices with control of the FDR at an allowed FDR of $10^{-3}$. (a) Example functional connectivity map using a seed in the left motor cortex. (b) Thresholds for significance using p-values generated by both Bartlett’s method (green) and xDF (red). Note that Bartlett’s method is more conservative than xDF, and no correlations were found to be significant by Bartlett’s method but not xDF. In this scan, overall 19.7% of correlations were significant by Bartlett’s method, and 29.6% were significant by xDF. In this image (a row of the overall correlation matrix), 43.1% of correlations are significant by Bartlett’s method and 52.7% by xDF. (c), (d) Data from the same run as above, now with a seed in the retrosplenial cortex. In this image (a row of the overall correlation matrix), 41.1% of correlations are significant by Bartlett’s method and 51.7% by xDF.

We then examined correlation matrices within a run (i.e., actual functional connectivity data with the correlations showing neuronal networks). Using the allowed FDR of 10^−3^ taken from the above analysis, the expected canonical network correlations and anticorrelations are outlined (Fig. 7). Using the p-values from Bartlett’s method resulted in a slightly more conservative threshold. Across all scans, using this FDR, xDF resulted in significantly more correlations meeting the threshold for statistical significance (xDF: median 22.3% of correlations, IQR: 17.8% to 26.2% versus Bartlett’s method: median 12.8% of correlations, IQR: 9.3% to 16.2%, p<0.0001). In no runs were there any individual pixels that were judged significant by Bartlett’s method but not by xDF.

**Fig. 8:**
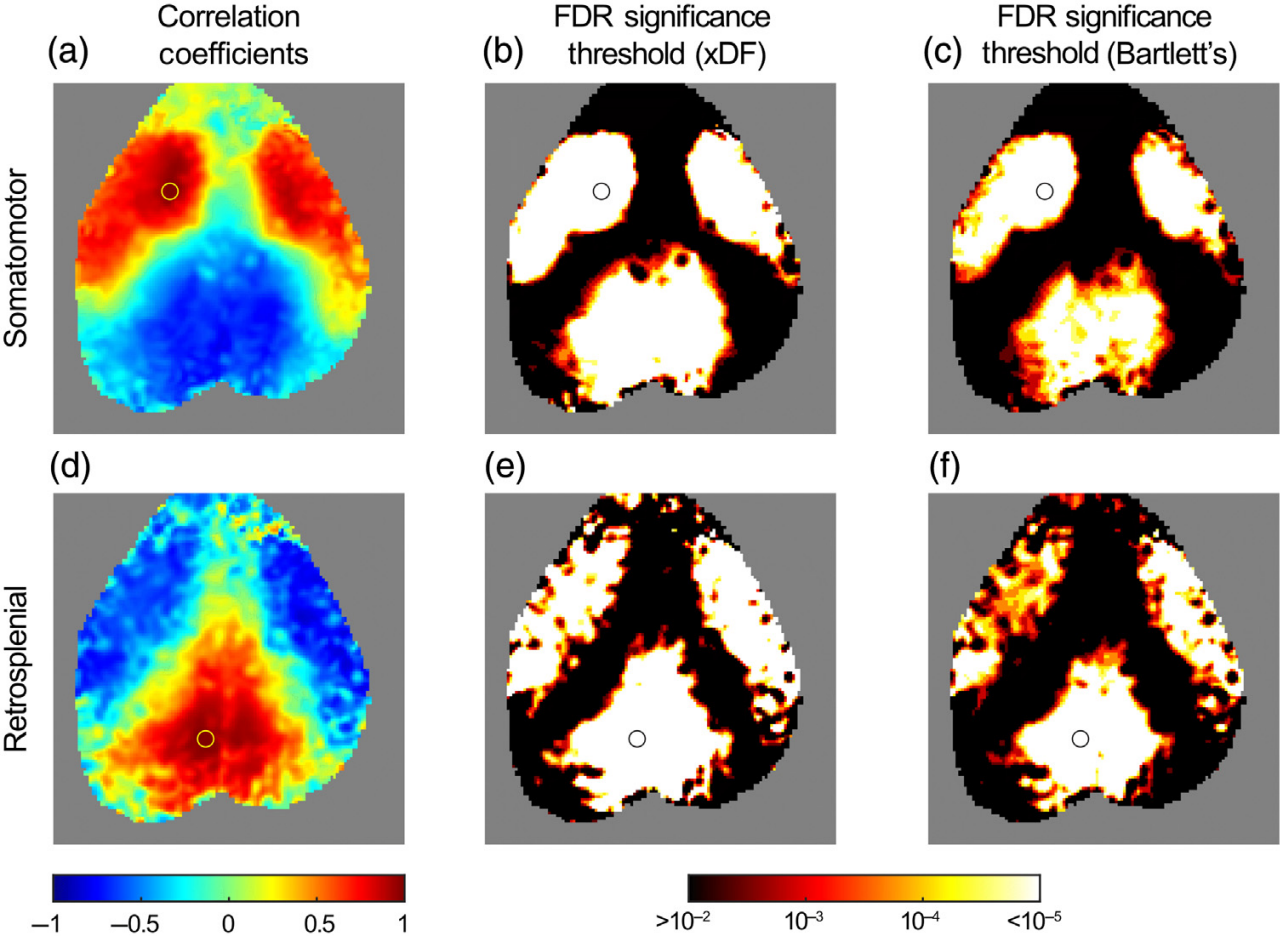
Effect of varying the allowed FDR on the thresholds for functional connectivity maps. (a) Example functional connectivity map using a seed in the left motor cortex. (b) Using p-values derived from xDF, each pixel is colored with the FDR at which that pixel is no longer judged significant. Black areas are where an FDR above $10^{-2}$ is required for statistical significance. White areas are where an FDR of $10^{-5}$ still results in significance. (c) Similar to (b) except using p-values derived from Bartlett’s method. (d)–(f) The same methods as in (a)–(c) now using a seed in the left retrosplenial cortex.

We then examined how the thresholding would behave with a range of acceptable FDRs. The boundary between significant and non-significant pixels was relatively independent of the exact allowable FDR chosen (Fig. 8). The core canonical regions of each network (and their anticorrelations) were preserved even to an FDR of $10^{-5}$. Similarly, even an FDR threshold of $10^{-3}$ did not result in significant expansion of the functional connectivity networks. This finding was true regardless of whether Bartlett’s method or xDF was used to generate the p-values.

## 4 Discussion

In this paper, we have proposed statistical methods for the interpretation of correlations in resting-state functional connectivity data from OIS imaging in mice. These methods account for the specifics of temporal and spatial autocorrelation seen in the data. Temporal autocorrelation arises from the hemodynamic response function, variations in systemic physiology (that survive global signal regression), and low-pass filtering of the data. Spatial autocorrelation arises from the point-response function of the diffuse optics, spatial smoothing of the data, and the fact that adjacent pixels represent neurologically similar regions of the brain. It is not possible in all cases to rigorously separate these contributions. But using methods to compensate for autocorrelation improves the rigor of interpreting which p-values represent statistical significance during a resting-state study.

In our analysis of temporal autocorrelation, we followed the analysis of Afyouni et al., who have provided the most complete account for the variance of correlation coefficients and demonstrated their theory with fMRI data in humans. Our findings differ from theirs in several interesting ways. We found less variation in the autocorrelation time across the cortex, and we found that averaging across regions of interest decreased the autocorrelation time and decreased spatial variation. Furthermore, the relationship between the size of the region of interest and the autocorrelation time was opposite that of Afyouni et al. It is possible that these differences arise due to differences between the human and mouse cortex. The mouse brain is simpler than the human brain with fewer regions devoted to high-level executive function. If the hemodynamic response function varied across cortical regions, driven by such complexity, then one might expect the mouse brain to be more homogenous. However, it is unclear if such a hypothesis would explain the results of Afyouni et al., in which variation was seen in cingulate, frontal, and somatosensory-motor cortices.

Alternatively, these differences between our autocorrelation time results and theirs may be driven by differences between the imaging systems. We are unaware of any studies comparing fMRI or optical methods in their ability to accurately resolve the true hemodynamic time course. However, it should be noted that OIS is not subject to many sources of variance found in fMRI, including magnetic field inhomogeneities or artifacts due to head motion. (However, we do note that we used two different cameras in the present study, each with its own noise characteristics, which may contribute to unintended variation.) It is possible that fMRI-specific confounders are partially responsible for the results of Afyouni et al. Although we are unable to provide a formal test of this fMRI hypothesis, the results of Afyouni et al. do demonstrate higher autocorrelation values in the periphery, where such artifacts are more likely. Finally, variance in the autocorrelation time may be due to partial volume effects in voxels/pixels containing partly brain and partly cerebrospinal fluid or skull. In our mouse data, we did see higher autocorrelation times in pixels at the periphery and close to venous sinuses. As the mouse brain is not folded into sulci and gyri, partial volume effects would be expected to be lower overall than in human fMRI.

When examining variance and the resulting *p*-values of the correlation coefficients, our findings are similar to Afyouni et al. Bartlett’s method of correction results in higher variance for high correlation values, which suppresses the resulting z scores. Conversely, for highly correlated pixels, xDF results in a smaller variance and z scores at or near the naïve limit. Overall, Bartlett’s method results in a distribution of z scores that is closer to the normal distribution under null hypothesis conditions than xDF. As Bartlett’s method assumes the autocorrelation time is homogenous within and across mice, this method may work better for OIS data in mice than in human fMRI.

We note that, in addition to the above approaches to account for temporal autocorrelation, another approach is to prewhiten data to remove temporal autocorrelations. Originally developed for fMRI, these techniques have been adopted for functional near-infrared spectroscopy (fNIRS).^27^^–^^30^ However, prewhitening flattens the power spectrum and removes temporal relationships between measurements, which may be problematic for low-frequency resting-state functional connectivity analysis and limits the ability to perform time-lag calculations or dynamic connectivity analyses. Additionally, prewhitening relies on the creation of an accurate autoregressive model for the data, which has not been performed for OIS; techniques developed for fMRI are likely not applicable due to the large differences in sampling frequency.

To address the problem of spatial autocorrelation, we elected to use inference methods to control the FDR that are robust to different correlation structures. This approach is in contrast to methods that control the FWER, which is too conservative applied across all pixels. The most common method to control the FWER in functional neuroimaging is the use of RFT,^13^ usually assuming a Gaussian random field, although analyses using other distributions are possible.^31^ Random fields have been used for human task-based imaging with fNIRS.^27^^,^^32^ Application of these techniques to resting-state functional connectivity data is difficult, as the statistical space (the correlation matrix) is now N(N−1), where N is the dimensionality of the images.^15^^,^^33^ The relevant parameter is the covariance of different regions in the correlation matrix; any individual column or row may be a random field, whereas the entire matrix may have a different excursion set.^34^ Additionally, the expected “roughness” of the random field is often calculated *post hoc* using experimental (rather than null hypothesis) data, and the roughness of an image is not equivalent to the roughness of the correlation matrix.

RFT is also highly dependent on the geometry of the brain images. As originally conceived, these methods assumed no activations touched the edge of the field of view.^35^ In OIS, as in many imaging modalities, this assumption is violated. Later extensions of RFT developed methods to account for a spatially restricted region.^14^ However, these corrections still assume that the imaged space is convex without holes; an assumption that may be violated in fMRI or OIS analysis. For example, in fMRI, the two hemispheres are often analyzed as two separated surfaces.^36^ Similarly, in OIS, the field of view may be divided into two separated hemispheres^37^^,^^38^ or interrupted by censored pixels.^20^ The multidimensional “resel” calculations of Worsley et al.^14^ do not fully account for the effects of these geometries on the resulting Euler characteristic. These are among the many reasons that make random field methods difficult to use in practice for OIS data.

In contrast, the methods used here to control the FDR have less strict assumptions about the spatial covariance, are computationally simpler, and are more widely applicable. The Benjamini–Hochberg and Benjamini–Yekutieli methods have been used for channel-wise fNIRS and task-based paradigm.^39^ However, we are not aware of its prior use in OIS data or in optical resting-state functional connectivity. We have demonstrated how these methods can be applied to correlation data in OIS. After controlling the temporal autocorrelation, it is possible to choose an allowed FDR that appropriately results in no significant correlations under null hypothesis conditions. When applied to typical experimental data, the resulting threshold maps highlight the expected canonical networks and their anticorrelations. This threshold is relatively independent of the FDR chosen, within a reasonable range.

Overall, our results demonstrate that use of Bartlett’s method and control of FDR provide a simple and robust method for determining statistical associations in resting-state functional connectivity data from OIS in mice. Although xDF may improve variance calculations in highly correlated regions, these differences are of minimal practical consequence in determining thresholds. In fact, Bartlett’s method results in a more conservative estimation of the functional connectivity network and excludes false positives more robustly under null hypothesis conditions. Additionally, Bartlett’s method is significantly less computationally intensive than xDF. Thus, in OIS, the theoretical benefits of xDF likely do not result in practical improvements that are worth these costs.

## 5 Code, Data, and Materials Availability

The software code used in the research will be made available for research use upon reasonable requests made to the corresponding author.
